# Mortality in the USA, the UK and Other Western Countries, 1989–2015: What Is Wrong With the US?

**DOI:** 10.1177/0020731420965130

**Published:** 2020-10-15

**Authors:** Colin Pritchard, Sam Porters, Emily Rosenorn-Lanng, Richard Williams

**Affiliations:** 1 Faculty of Health and Social Sciences, Bournemouth University, Bournemouth, UK

**Keywords:** international, comparison, mortality, USA, Western world, health, expenditure

## Abstract

This population-based study compares U.S. effectiveness with 20 Other Western Countries (OWC) in reducing mortality 1989–1991 and 2013–2015 and, responding to criticisms of Britain’s National Health Service, directly compares U.S. with U.K. child (0–4), adult (55–74), and 24 global mortality categories. World Health Organization Age-Standardized Death Rates (ASDR) data are used to compare American and OWC mortality over the period, juxtaposed against national average percentages of Gross Domestic Product (GDP) Expenditure on Health (%GDPEH) drawn from World Bank data. America’s average %GDPEH was highest at 13.53% and Britain’s the lowest at 7.68%. Every OWC had significantly greater ASDR reductions than America. Current U.S. child and adult mortality rates are 46% and 19% higher than Britain’s. Of 24 global diagnostic mortalities, America had 16 higher rates than Britain, notably for Circulatory Disease (24%), Endocrine Disorders (70%), External Deaths (53%), Genitourinary (44%), Infectious Disease (65%), and Perinatal Deaths (34%). Conversely, U.S. rates were *lower* than Britain’s for Neoplasms (11%), Respiratory (12%), and Digestive Disorder Deaths (11%). However, had America matched the United Kingdom’s ASDR, there would have been 488,453 fewer U.S. deaths. In view of American %GDPHE and their mortality rates, which were significantly higher than those of the OWC, these results suggests that the U.S. health care system is the least efficient in the Western world.

In the recent debate about repealing the Affordable Health Care Act (“Obamacare”), it was claimed that Obamacare was more expensive and that state intervention invariably entailed greater inefficiencies than a free market approach to health care.^1^ In that debate, the United Kingdom’s National Health Service (NHS) system was critically singled out as an exemplar of the ineffectiveness of what was described as “socialized” health care, exemplified by the fact that U.K. cancer survival and cancer mortality rates were higher than those of the United States.^2–4^

Trying to compare countries’ health care systems raises the problem of how to measure the effectiveness of any nation’s health system. We argue that one key health-related outcome is reducing amendable mortality rates. This reflects the UNICEF statement “that in the last analysis child *mortality rates* (our emphasis) are an indicator of how well a nation meets the needs of its children.”^5^ We transpose this concept by stating that amendable mortality rates are indicative of how well a nation meets the health needs of its population.

Thus does the United States, the richest country in the world, achieve as good a return for its health investment by reducing its mortality as well as or better than other nations?

From an economic perspective, it might be assumed there would be a link between a nation’s financial investment in health and its reduction in mortality rates, the classic “you get what you pay for.” However, it has long been known that the United States spends a far higher percentage of Gross Domestic Product (GDP) Expenditure on Health (%GDPEH) than any other country.^6–8^ Indeed, American researchers Himmelstein and Woolhandler, long-term critics of the U.S. system, argue that the U.S. private insurance multi-payer system is inefficient and over-burdened with administrative costs and that, despite Obamacare, there continues to be considerable unmet need.^9–13^ So, relatively, how effective is the American health care system?

The answer lies in comparing the United States’ and the 20 Other Western Countries’ (OWC) World Health Organization (WHO)-based mortality rates^13^ and their %GDPEH, based on World Bank data.^6^ In addition, in response to the criticism of “state intervention” as exemplified by the United Kingdom’s NHS, we make a direct comparison of current health outcomes for each of the 24 major global mortality categories between the United Kingdom and America.

Three specific measures will be used. World Bank data, extrapolating from 1980 each country’s %GDPEH to the latest year, including all 27 recorded years, ending in 2016 from which an average %GDPEH is calculated.^6^ To illustrate changes over time, total %GDPEH levels are given for 2000, 2010, and 2016 and for the percentage of GDPEH that comes from “private” sources, provided mainly by individuals or employment-based insurance. This “private” element is included in the total %GDPEH of each nation, as all have a proportion of their total %GDPEH that comes from “private.”^6^

The “clinical” outcomes are total mortality rates based on the WHO world population Age-Standardized Death Rates (ASDR) reported here in rates per million (pm),^13^ which are used to compare the United States with the 20 OWC.

Third, in answer to criticisms of the British NHS, we undertake a direct comparison between the U.S. and the U.K. current child mortality rates (CMR 0–4 years) and adult mortality rates of people aged 55–74 (AMR 55–74 years), which is below the average life expectancy of Western countries.^13^ Thus, this age band can be considered as amendable mortality for all Western nations following health care improvements. In addition are the 24 selected global mortalities categories listed in the U.S. National Vital Statistics report.^14^ We use these 24 categories to undertake a detailed comparison of current American and British mortality rates.^13^

It is recognized that there are confounding influences that affect health outcomes other than a nation’s expenditure, such as pre-existing social determinants; a range of public health and socioeconomic issues, such as access to health care,^15–17^ and the continuing problem of a significant proportion of U.S. citizens who are not insured or underinsured.^17–19^ The strength of this study is that is brings together issues that are usually examined separately and, crucially, this will be the first direct comparative analysis of British and American amendable mortality outcomes of predominantly “private” vs “public” health-funded countries.^6^

The 3 null working hypotheses are that between 1989–1991 and 2013–2015, there will be no significant differences in reducing amendable mortality:

1. between the United States and the OWC in reducing Age-Standardized Death Rates (ASDR)
2. between the United States and the United Kingdom in reducing child (0–4) or adult (55–74) mortality
3. between the U.S. and U.K. death rates of each of the 24 major global mortality categories.^14^

## Methodology

All mortality rates are drawn from WHO annual mortality data, updated December 2018, to compare the United States and the 20 OWC on the WHO’s world population Age-Standardized-Death-Rates (ASDR) per million (pm), which are total death rates controlled for population and age, using a standardized method adjusting for population demographics.^13^ The baseline is a 3-year average of 1989–1991 compared to average index years for 2013–2015 average, which are the latest available years. Germany’s baseline years were 1990–1992 and 4 countries (Canada, New Zealand, France, and Ireland) have earlier index years (2011–2013 or 2012–2014), are noted in the tables. Each country acts as its own control by being compared with its own baseline and index years, from which a ratio of change is calculated.

### Comparing the United Kingdom and United States Current Mortalities

The direct comparisons between the United States and the United Kingdom are for current child mortality rates (CMR 0–4), based on the WHO age bands > 1 year and 1–4 years, from which a mortality rate pm for the CMR 0–4 is calculated. Adult mortality rates (AMR) for people aged 55–74 years are based on the numbers of total deaths in the 55- to 74-year-old population to calculate AMR pm.^13^

The 24 major global mortality categories were examined where there was a death rate reaching at least 10 pm in either the United Kingdom or the United States.^13^ This resolves any issues of differential causes of death, as the focus is only on global categories, which cover a number of separate diagnoses. For example, the global Circulatory Disease Deaths category includes ischemic heart disease, cerebrovascular disease, hypertension, rheumatic hearts disease, and arterial disease-related deaths. The 10th edition of the International Classification of Diseases began in 1989 and is used by the WHO to define global diagnostic categories, hence our baseline years.^13^

It should be noted that for the global category External Deaths, we report on each separate diagnostic group because of their frequency and because they reflect *psychosocial factors and lifestyles in society*. These include suicide and assault deaths and “Undetermined deaths.”^5,14,15,20–23^ The latter group occurs when authorities cannot determine whether the death was accidental, was self-inflicted, or included a third party. But all involve a degree of lethality similar to suicidal methods of death;^13^ accordingly, “undetermined deaths” and “accidental poisoning” are examined as they are thought to be a source of likely underreported suicide.^23^

The economic input into health care is the average national percentage of %GDPEH drawn from the World Bank (updated July 2019), based on all years between 1980 and 2016.^6^ Thus, %GDPEH can be seen as a general measure of the financial priority each nation gives to health and social care, quite irrespective of how the services are funded or configured.

The World Bank reports on 2 sources of GDP funding for health. These are “Public,” which come from federal/national and state sources, and “Private,” which comes from individual or employment-linked health insurance plans. The “Public” and combined “Private” percentages are the total %GDPEH of each nation.^6^ Total %GDP is given for the years 1980, 2000, 2010, and 2016 to illustrate the changes over the period. For reasons of space, only the %GDP coming from “Private” sources is shown for the latest year available (2016). It should be noted that every country has made substantial increases in %GDPEH since 1980. However, it is known that the United Kingdom has long had one of the lowest %GDP devoted to health and the United States one of the highest.^4,6–9,13^ A ratio of change is calculated for each OWC average %GDPEH and compared to the U.S. average, from which an odds ratio is calculated. Finally, the percentage of GDPEH that comes from “Private” sources is shown to give an indication of the “Public/Private” funding configuration of each country.^6^

The data commenced in 1980 and continued to 2016, but no data was reported for the years 1982, 1983, 1985, 1992, 1996, 2002, 2003, 2006, 2007, and 2009. In addition, there was no %GDPEH-reported data for Australia in 2004 and 2008, for Belgium in 2004, for Denmark in 2008, for Germany in 2004, for Greece in 1994, 2004, and 2008, and for Japan in 2004 and 2008.^6^ The numbers of missing years for each country are indicated in Table 1.

**Table 1.** Total % GDP Expenditure on Health 1980–2016. OWC vs U.S. Current Government % Health Expenditure and U.S. average %GDP to OWC Odds Ratios.

Country and Average Rank	Total GDP 1980	Total GDP 2000	Total GDP 2010		Total GDP 2016	Total Average 1980–2016	OWC: U.S. %GDPEH Ratio
**1. U.S.**	**9.0**	**13.4**	**17.1**		**17.07**	**13.53**	**1:1.00**
2. Switzerland	7.3	10.2	10.9		12.25	10.17	1:1.34
3. Germany [1]	8.4	10.1	11.6		11.14	10.16	1:1.33
4. France	7.0	10.3	11.6		11.54	10.13	1:1.34
5. Canada	7.0	8.8	11.1		10.53	9.82	1:1.38
6. Austria [2]	7.4	9.0	11.1		10.44	9.64	1:1.40
7. Belgium [1]	6.3	9.0	10.6		10.04	9.32	1:1.45
8. Denmark [1]	8.9	8.3	11.1		10.3	9.10	1:1.49
9. Netherlands	7.4	8.0	12.1		10.36	9.08	1:1.49
10. Sweden	8.9	8.2	9.5		10.93	8.85	1:1.53
11. Norway	7.0	8.4	9.4		10.0	8.55	1:1.58
12. Australia [2]	6.1	8.0	8.9		9.25	8.46	1:1.60
13. New Zealand	5.9	7.7	10.0		9.22	8.37	1:1.62
14. Japan [2]	6.5	7.7	9.6		10.93	8.34	1:1.62
15. Finland	6.3	7.2	9.0		9.49	8.32	1:1.63
16. Italy	7.0	8.1	9.4		8.94	8.15	1:1.66
17. Portugal [1]	5.3	8.8	10.9		9.08	8.13	1:1.66
18. Ireland	8.2	6.1	9.2		7.38	8.03	1:1.68
19. Spain	5.3	7.2	9.6		9.2	7.77	1:1.74
20. Greece [3]	5.9	7.9	9.5		8.4	7.71	1:1.75
**21. U.K**	**5.6**	**7.0**	**9.4**		**9.9**	**7.68**	**1:1.76**
**OWC (-U.S.) average**	**6.8**	**8.3**	**10.2**		**9.97**	**8.79**	**8.81**
**OWC: U.S. ratios**	**1:1.32**	**1:1.61**	**1:1.68**		**1:1.71**	**1.54**	**1:1.54**

Abbreviations: GDP, gross domestic product; GDPEH, GDP expenditure on health (%); OWC, other Western countries.

Missing years in brackets. Bold values highlight US and UK expenditures.

An inherent problem of international comparisons is how different health and social policies and configurations of health services might influence health outcomes. This would require country-specific research to resolve this problem and is outside the scope of this study. Nevertheless, the %GDPEH is a reasonable measure of the fiscal priority each country gives to its health and social care services.

### Statistics

We used the SPSS statistical package from which Confidence Intervals (to ±95% significance) are calculated to compare changes in ASDR over the period between the United States and the OWC, all controlled for total population.^13^ The comparison between U.S. and U.K. current child (0–4) and adult (55–74) mortalities and the 24 global mortality categories are based on the average of years 2013–2015 in rates pm. A series of U.K. to U.S. odds ratios are calculated for each of the current rates of the global diagnostic mortality categories.

## Results

### %GDP Expenditure on Health

Table 1 presents changes in %GDPEH between 1980 and 2016. Over the period, the United States always had the highest current and average %GDPEH (17.07% and 13.53%, respectively). Apart from America, only France, Germany, and Switzerland averaged above 10% of GDP being devoted to health care over the period. The lowest average %GDPEH was the United Kingdom at 7.68%, followed by Greece at 7.71% and Spain at 7.77%, with the overall OWC average being 8.79%.

The fifth column of Table 1 lists the percentage of GDP on health coming from “Private” sources. Unsurprisingly, the United States has the highest “Private” input at 9.15%, followed by Switzerland at 4.8% and the Netherlands at 4.02%. The lowest proportion of %GDP coming from “Private” sources was Denmark at 1.33%, Norway at 1.53%, and the United Kingdom at 1.58%, with an OWC average of 2.09% and the remaining 6.7% of national GDP coming from “Public” sources.

The *proportion* of total health expenditure coming from “Private” sources was more varied than expected, as only 54% of total American %GDPEH came from “Private” sources, meaning there were considerable funds via federal and state funding. There were highs of 40% from “Private” sources in Australia and Netherlands and in Switzerland 39% contributing to total %GDPEH. The “lowest” proportions of “Private” contributions to total %GDPEH were found in Denmark at 13%, Norway at 15%, and the United Kingdom at 16%. The OWC average “Private” source was 24%.

The odds ratio of OWC and United States of average %GDPEH was 1:1.54 over the whole period, with 12 countries having ratios of wider ratios than > 1:1.50. As the United Kingdom had the lowest average %GDPEH of all countries reviewed, the widest odds ratio was between Britain and America at 1:1.76.

It is noteworthy that in the specific years shown (1980, 2000, 2010, and 2016), U.S. spending on health rose in proportion to what the OWC were spending, as the ratio in 1980 was 1:1.32 but by 2016 had risen to 1:1.71, indicating that over the period, the United States was relatively increasing its %GDPEH compared to the OWC.

### Comparing Other Western Countries to U.S. Age-Standardized Death Rates

Table 2 presents the changes in total mortality rates pm between 1989–1991 and 2013–2015. The United States had the highest current ASDR at 4,772 pm, followed by Germany at 4,173 pm and Portugal at 4,136 pm. The lowest ASDR were in Japan at 3,084 pm, Switzerland at 3,326 pm, and Australia at 3,420 pm.

**Table 2.** Age-Standardized Deaths All Cause Deaths per Million 1989–1991 vs 2013–2015: CIs Used to Compare OWC With U.S.

Country, Ranks Year 2013–2015 vs 1989–1991	ASDR1989–1991	ASDR2013–2015	% change	OWC vs U.S. Ratio	CILower	CI Upper
**1-9. U.S.**	**6,203**	**4,772**	**−23%**	1:1.00	1:1.00	1:1.00
2-5. Germany	6,456	4,173	−35%	1:1.19	1:1.13	1:1.26
3-2. Portugal 2012–2014	7,086	4,136	−42%	1:1.32	1:1.25	1:1.39
4-4. Denmark	6,512	4,124	−37%	1:1.21	1:1.15	1:1.28
5-10. Belgium	6,151	4,081	−34%	1:1.16	1:1.10	1:1.22
6-1. Ireland 2012–2014	7,127	4,065	−43%	1:1.35	1:1.28	1:1.42
**7-6. U.K.**	**6,340**	**4,044**	**−36%**	**1:1.21**	**1:1.14**	**1:1.27**
8-13. Greece	5,694	3,995	−30%	1:1.10	1:1.04	1:1.16
9-3. Finland	6,613	3,985	−40%	1:1.28	1:1.21	1:1.35
10-7. Austria	6,334	3,945	−38%	1:1.24	1:1.17	1:1.3
11-12. Netherlands	5,739	3,898	−32%	1:1.13	1:1.07	1:1.2
12-8. New Zealand 2011–2013	6,278	3,879	−38%	1:1.25	1:1.18	1:1.32
13-11. Norway	5,862	3,697	−37%	1:1.22	1:1.15	1:1.29
14-18. Sweden	5,420	3,688	−32%	1:1.13	1:1.07	1:1.2
15-17. Canada 2011–2013	5,504	3,663	−33%	1:1.16	1:1.09	1:1.22
16-19. France 2012–2014	5,377	3,604	−33%	1:1.15	1:1.08	1:1.21
17-14. Italy	5,675	3,455	−39%	1:1.26	1:1.19	1:1.34
18-15. Spain	5,670	3,422	−40%	1:1.27	1:1.20	1:1.35
19-16. Australia	5,641	3,420	−39%	1:1.27	1:1.20	1:1.34
20-20. Switzerland	4,930	3,326	−33%	1:1.14	1:1.08	1:1.21
21-21. Japan	4,675	3,084	−34%	1:1.17	1:1.10	1:1.24
**Average (-U.S.)**	5,735	3,784	−34%			

Abbreviations: ASDR, age-standardized death rate; CI, confidence interval; OWC, other Western countries.

The United States had the smallest reduction in ASDR at just 23% over the period, compared to an average 34% decline in the OWC. The United Kingdom was seventh highest at 4,044 pm, which represented a drop of 36%, meaning that American ASDR were 18% higher than the United Kingdom.

Confidence Interval analysis showed that every Western country had a statistically significant larger reduction in total mortality than the United States over the period.

### Comparing United Kingdom to United States on 24 Mortality Categories

Table 3 presents the differences between current U.K. and U.S. mortality for children (0–4) and adults (55–74) and for the 24 global mortality categories.

**Table 3.** U.K. Compared to U.S., Child and Adult Mortality and Current Global Diagnostic Death Rates per Million (pm) > 10 pm. 2013–2015.

*Category-U.K. lower than U.S.*	U.K.	U.S.	U.K.: U.S. Ratio
**Child mortality (0–4 years)**	**855pm**	**1,249 pm**	**1:1.46**
**Adult mortality (55–74 years)**	**10,568 pm**	**12,544 pm**	**1:1.19**
1. Perinatal deaths (< 1year)	2,066pm	3,113pm	1:1.51
2. Circulatory disease deaths	1,010 pm	1,326 pm	1:1.31
3. Nervous disease	188pm	256pm	1:1.36
4. Alzheimer	309pm	308pm	1:1.00
Total neurological deaths	497pm	564pm	1:1.13
5. Accidental poisoning	59pm	148pm	1:2.51
6. Suicide	67pm	120pm	1:1.79
7. Transport	26pm	110pm	1:4.23
8. Falls	98pm	104pm	1:1.06
9. Assault	2pm	55pm	1: 27.5
10. Drowning	5pm	11pm	1:2.13
Total external deaths	257pm	548pm	1:2.13
11. Endocrine disorders	63pm	213pm	1:3.38
12. Infectious diseases	47pm	136pm	1:2.89
[includes HIV]	2pm	18pm	1:9.00
13. Genitourinary disorders	61pm	108pm	1:1.79
14. Congenital conditions	33pm	37pm	1:1.12
15. Blood and immune disorders	10pm	20pm	1:2.00
16. Pregnancy and childbirth-female	4pm	14pm	1:3.50
*Category U.K. higher than U.S.*			
17. Neoplasms	1,273 pm	1,138 pm	1:0.89
18. Respiratory	496pm	438pm	1:0.88
19. Mental and behavioral	242pm	202pm	1:0.83
20. Digestive disorders	221pm	197pm	1:0.89
21. Undetermined deaths [external]	102pm	87pm	1:0.85
22. Problem signs and symptoms (< 1)	68pm	67pm	1:0.99
23. Skeletal and connective	29pm	25pm	1:0.86
24. Skin	13pm	8pm	1:0.62

America’s child mortality was 1,249 pm compared to the United Kingdom’s 885pm, which means that U.S. rates were 46% higher than Britain’s.

U.K. adult (55–74) rates were 10,568 pm to the U.S. 12,544 pm, which were 19% higher than the United Kingdom’s adult rate.

In 2015, the latest year for which we have WHO mortality data, total numbers of U.S. deaths were 2,713,630. Thus, if America had matched Britain’s ASDR in 2015, there would have been 488,453 fewer U.S. deaths, including 10,789 fewer child deaths (0–4).

America had substantially higher rates than Britain for 16 categories of the 24 mortality categories.

These were as follows: U.K. to U.S. Perinatal Deaths (2,066 pm to 3,113 pm), Circulatory Disease Deaths (1,010 pm to 1,326 pm), Total Neurological (497pm to 564pm), Total External Deaths (257pm to 548pm), Endocrine Disorders (63pm to 213pm), Infectious Diseases (47pm to 136pm), Genitourinary Disorders (61pm to 108pm), Congenital Conditions (33pm to 37pm), Blood and Immune Disorders (10pm to 20pm), and deaths surrounding childbirth (4pm to 14pm).

Substantial U.K. to U.S. odds ratios (> 1:1.20) for mortalities reaching at least 100pm were Endocrine Disorder Deaths (1:3.41), Infectious Disease (1:2.93), Genitourinary (1:1.79), Perinatal (1:1.51), Circulatory Disease (1:1.31), and Neurological Disease Deaths (1:1.13), which include such conditions as motor neurone disease, Parkinson’s disease, and multiple system atrophy, but not Alzheimer’s and other dementias, which were virtually identical in the 2 countries. Notable total external deaths included suicide, yielding U.K. to U.S. ratios of (1:1.79), transports deaths (1:4.23), assaults (1:27.5), and accidental poisoning (1:2.51), which is associated with underreported suicides.

Conversely, there were 5 substantial mortality category death rates (> 100pm) in which Britain had *higher* rates than America: Neoplasms (1,273 pm to 1,138 pm, yielding a U.K. to U.S. odds ratios of 1:0.89), Respiratory Diseases (496pm to 438pm, a 1:0.88 ratio), Mental and Behavioral Disorders (242pm to 203pm, a 1:0.83 ratio), Digestive Disorder Deaths (221pm to197pm, a1:0.89 ratio), and Undetermined Deaths (102pm to 87pm, a 1:0.85 ratio).

In addition, the United Kingdom had slightly higher rates than America for Problematic Signs and Symptoms (68pm to 67pm), Skeletal and Connective (29pm to 25pm), and Skin-Related Deaths (13pm to 8pm).

## Discussion

It is readily acknowledged that a complex range of interactive, multiple causes influence national mortality rates,^15–17^ but relative poverty is especially linked to child mortality, although the underlying mechanism is not fully understood.^15–17,24–27^ Lifestyles and what might be described as the local “geography” of American states are other factors.^27–29^ However, placing these results within the context of each nation’s fiscal investment to health poses the question of comparative effectiveness of the different countries. Although %GDPEH is but one aspect of health care delivery, it is the major economic indicator of a nation’s fiscal commitment to the health needs of its population.

One surprising finding was that only 54% of total U.S. health expenditure came from “Private” sources, which highlights the considerable funds coming from federal and state provision, yet as the U.S. system is still based on an insurance model, there remains the problem of limited or underinsured people not having their health needs met.^1,6,11,16–18,24,25^ Indeed, it has long been known that American health is more expensive in part because of over-prescription, overuse of high-tech measures, and higher administrative costs—in effect, “spending more and getting less.”^6,9–11,30–34^ Although it has been recognized that the not-for-profit systems are less costly,^33^ it is still being argued that the Obama Reform was “no cure for what ails us”;^35^ although this is possibly complicated by interactive socioeconomic factors,^14–18,24–27^ it is predominately because health care is delivered via an insurance system that needs to make a profit.^7,32,33^

The 3 null hypotheses are rejected as the United States had significantly higher mortality rates than the 20 OWC and, compared to the United Kingdom, had significantly higher rates for child and adult mortality and for most of the global diagnostic categories. Indeed, as all the other countries had significantly lower ASDR than America, this suggests that the U.S. health care system is the least efficient of the 20 Western nations in reducing amendable mortality. This study also shows that of the specific mortality categories, the United Kingdom had lower rates in 16 of the 24 categories, and had American matched the United Kingdom’s overall mortality rates, there would have been 488,433 fewer American deaths. This raises questions as to whether the money spent on health reaches the patient, as America’s failure to match Britain’s total mortality suggests that the United States does not get a good “lifesaving” return on its health care investment.

We can only briefly speculate on possible factors that might have contributed to the key differences between the U.K. and U.S. results.

The 6 biggest global mortality rates for which America exceeds Britain possibly reflect something of the lifestyle and culture of the United States. The problems of obesity appear to be reflected in the very high U.S. circulatory and endocrine disorder deaths, which include diabetes.^36^ Then there is the issue of relative poverty, as the United States has long had the widest income inequality of all 21 Western countries, which might be reflected in the high infectious disease and perinatal deaths, as again the United States has long had the highest rates of child mortality (0–4) in the OWC, which is related to relative poverty.^15–17,25–27,37^ The differential results in HIV and external deaths—especially the higher suicide, transport, and assault deaths—appear to mirror differing societal lifestyles. One notable difference between the other nations is the extent of gun-related deaths in America linked to suicide and homicide,^38,39^ conservatively estimated at more than 30,000 p.a., which far exceeds any other Western country.^40^

Finally, based on the most authoritative data sources (namely the WHO and World Bank), this study shows that the predominantly universal British NHS compares very favorably to the United States, as America achieved proportionately less with relatively more, whereas the United Kingdom achieved proportionally more with relatively less. This gives support to the American policy analysts who asked, “Is American health care uniquely inefficient?”^34^

Whatever the answer to this question, attempts by opponents of the Affordable Health Care Act to portray the U.K. health care system as an exemplar of the ineffectiveness of “socialized health” is without empirical merit, and Britain’s substantially lower death rate compared to that of the United States should be a boost for frontline NHS staff, patients, and families.
